# Mast cell‐based molecular subtypes and signature associated with clinical outcome in early‐stage lung adenocarcinoma

**DOI:** 10.1002/1878-0261.12670

**Published:** 2020-04-01

**Authors:** Xuanwen Bao, Run Shi, Tianyu Zhao, Yanfang Wang

**Affiliations:** ^1^ Institute of Radiation Biology Helmholtz Center Munich German Research Center for Environmental Health Neuherberg Germany; ^2^ Technical University Munich (TUM) Germany; ^3^ Department of Radiation Oncology University Hospital Ludwig Maximilian University of Munich Germany; ^4^ Institute and Clinic for Occupational, Social and Environmental Medicine University Hospital Ludwig Maximilian University of Munich Germany; ^5^ Comprehensive Pneumology Center (CPC) Munich, Member DZL Germany; ^6^ German Center for Lung Research Munich Germany; ^7^ Institute of Epidemiology Helmholtz Zentrum München German Research Center for Environmental Health Neuherberg Germany; ^8^ Ludwig‐Maximilians‐Universität München (LMU) Munich Germany

**Keywords:** early‐stage lung adenocarcinoma, immunotherapy, mast cell, prognosis

## Abstract

Mast cells are a major component of the immune microenvironment in tumour tissues and modulate tumour progression by releasing pro‐tumorigenic and antitumorigenic molecules. Regarding the impact of mast cells on the outcomes of patients with lung adenocarcinoma (LUAD) patient, several published studies have shown contradictory results. Here, we aimed at elucidating the role of mast cells in early‐stage LUAD. We found that high mast cell abundance was correlated with prolonged survival in early‐stage LUAD patients. The mast cell‐related gene signature and gene mutation data sets were used to stratify early‐stage LUAD patients into two molecular subtypes (subtype 1 and subtype 2). The neural network‐based framework constructed with the mast cell‐related signature showed high accuracy in predicting response to immunotherapy. Importantly, the prognostic mast cell‐related signature predicted the survival probability and the potential relationship between TP53 mutation, c‐MYC activation and mast cell activities. The meta‐analysis confirmed the prognostic value of the mast cell‐related gene signature. In summary, this study might improve our understanding of the role of mast cells in early‐stage LUAD and aid in the development of immunotherapy and personalized treatments for early‐stage LUAD patients.

## Graphical abstract

Mast cell abundance and a mast cell‐related signature were correlated with survival in early‐stage lung adenocarcinoma patients. The mast cell‐related signature‐based neural network showed high accuracy in predicting response to immunotherapy.

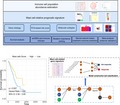

**Abbreviations**
DEG: differently expressed gene
GEO: Gene Expression Omnibus
GO: gene ontology
GSEA: gene set enrichment analysis
HR: hazard ratio
LUAD: lung adenocarcinoma
PCA: principal component analysis
ssGSEA: single‐sample gene set enrichment analysis
TCGA: The Cancer Genome Atlas
WGCNA: weighted correlation network analysis

## 1 Introduction

Lung adenocarcinoma (LUAD) is one of the most complex and heterogeneous malignancies, both in molecular and phenotypic terms (Li *et al.*, 2016; Mao *et al.*, 2016; Wang *et al.*, 2019a). The incidence of LUAD has been increasing in recent years. The treatment for early‐stage LUAD includes operation, chemotherapy and radiotherapy (Besse *et al.*, 2015). Additionally, immunotherapy serves a promising therapeutic strategy in many cancer types (Bao *et al.*, 2019b; Couzin‐Frankel, 2013; Purwar *et al.*, 2012; Schumacher and Schreiber, 2015; Wasiuk *et al.*, 2012) as well. However, there is still a long way to go for immunotherapy in LUAD. A clear understanding of the tumour immune microenvironment may aid in the development of immunotherapy for LUAD patients.

Mast cell is widely distributed in different tissues and is a major component of the immune microenvironment in tumour tissues. Mast cells modulate tumour initiation and progression through the secretion of pro‐tumorigenic and antitumorigenic molecules (Varricchi *et al.*, 2017). The controversial roles of mast cells result in conflicting effects among different tumour types (Alì *et al.*, 2009; Carlini *et al.*, 2010; Gounaris *et al.*, 2007; Jeong *et al.*, 2013; Nordlund and Askenase, 1983; Sinnamon *et al.*, 2008; Welsh *et al.*, 2005; Yang *et al.*, 2011). Regarding the impact of mast cells on LUAD patient clinical outcomes, several contradictory results have been published (Carlini *et al.*, 2010; Imada *et al.*, 2000; Kurebayashi *et al.*, 2016; Li *et al.*, 2018; Nagata *et al.*, 2003; Takanami *et al.*, 2000). Although the results of these studies are quite different with respect to the prognostic values of mast cells, previous studies have shown that mast cell infiltration is more intensive in well‐differentiated tumours and low‐grade histologic subtypes than in poorly differentiated and high‐grade subtypes (Carlini *et al.*, 2010; Nagata *et al.*, 2003). Thus, in this study, we focused on the effect of mast cells in early‐stage LUAD patients. We analysed the potential role of mast cell, mast cell‐related genes and immunotherapy outcomes in early‐stage LUAD using bioinformatics models and machine learning methods.

## 2 Method

### 2.1 Data processing

The Cancer Genome Atlas (TCGA) transcriptome data, mutation data and clinical information were downloaded *via* the UCSC Xena Browser (https://xenabrowser.net/). http://www.ncbi.nlm.nih.gov/geo/query/acc.cgi?acc=GSE11969, http://www.ncbi.nlm.nih.gov/geo/query/acc.cgi?acc=GSE13213, http://www.ncbi.nlm.nih.gov/geo/query/acc.cgi?acc=GSE29013, http://www.ncbi.nlm.nih.gov/geo/query/acc.cgi?acc=GSE30219, http://www.ncbi.nlm.nih.gov/geo/query/acc.cgi?acc=GSE31210, http://www.ncbi.nlm.nih.gov/geo/query/acc.cgi?acc=GSE37745, http://www.ncbi.nlm.nih.gov/geo/query/acc.cgi?acc=GSE42127, http://www.ncbi.nlm.nih.gov/geo/query/acc.cgi?acc=GSE50081 and http://www.ncbi.nlm.nih.gov/geo/query/acc.cgi?acc=GSE72094 were downloaded from the Gene Expression Omnibus database (http://www.ncbi.nlm.nih.gov/geo/). The detailed TCGA clinical information is summarized in Table 1 and Appendix S1.

**Table 1:** Patient information.

Variable	Number
Gender (female/male)	166/142
TNM stage (stage I/stage II)	212/96
Lymph node metastasis (positive/negative)	61/247
Age (> 60/≤ 60/missing)	221/78/9
KRAS status (WT/MUT)	210/98
EGFR status (WT/MUT)	271/37
Smoking (no/yes/missing)	15/93/200

### 2.2 Estimation of the abundance of immune cell populations and implementation of weighted correlation network analysis

Transcriptome file of TCGA early‐stage LUAD was applied on xCell to estimate the abundance of different immune cell populations (Aran *et al.*, 2017; Newman *et al.*, 2015). Weighted correlation network analysis (WGCNA) was accomplished with the r package ‘WGCNA’ (Bao *et al.*, 2019a; Langfelder and Horvath, 2008; Wang *et al.*, 2019b). The expression profile of immune‐related gene (from https://www.innatedb.com/redirect.do?go=resourcesGeneLists) was applied as the input of WGCNA. Gene significance quantified the association of individual genes with mast cell density, and module membership represented the correlation between module eigengenes and gene expression profiles. A power of β = 3 and a scale‐free *R* ^2^ = 0.95 were set as soft‐threshold parameters to ensure a signed scale‐free co‐expression gene network. A total of six nongrey modules were generated. Among these modules, the yellow module depicting the highest correlation (*r* = 0.92, *P* = 4.2e‐115) was considered the most correlated with mast cell density. Survival analysis was performed using the r package ‘survival’. Cox regression analysis was used to determine the hazard ratio (HR). All genes in the yellow module were subjected to univariate Cox regression. The 110 genes that significantly associated with the survival of early‐stage LUAD patients in the yellow module were identified as the mast cell‐related gene signature. These identified genes were applied on gene ontology (GO) analysis with the ‘clusterProfiler’ package (Yu *et al.*, 2012) to elucidate the potential mechanism behind the gene signature. r software (version: 3.5.3) was used for all the analyses in the manuscript.

### 2.3 Molecular subtype identification

The r package ‘CancerSubtypes’ was applied to perform molecular subtype identification (Xu *et al.*, 2017). Transcriptome profile and gene mutation data sets were used to perform cancer subtype analysis. The default parameters were used to perform the classification. The cluster number was selected as 2. Gene set enrichment analysis (GSEA) was performed with GSEA software from Broad Institute.

### 2.4 Differently expressed gene analysis

The differently expressed gene (DEG) analysis was performed with ‘Limma’ package (Smyth, 2005). An empirical Bayesian method was applied to estimate the fold change between the molecular subtype 1 and 2 using moderated *t*‐tests. The adjusted *P*‐value for multiple testing was calculated using the Benjamini–Hochberg correction. The genes with an adjusted *P*‐value < 0.05 and absolute log_2_ (log to base two of) fold change > 1.5 were identified as DEGs between two molecular subtypes. GO analysis was performed based on the significant genes.

### 2.5 Prognostic gene signature‐based risk score and ssGSEA implementation

The genes in the WGCNA yellow module were analysed with univariate Cox regression analysis. The comprehensive mast cell‐related signature was calculated by principal component analysis (PCA). The PCA‐based risk score MastCell_pca_ was derived from the first principal component of the 110 genes from mast cell‐related gene signature. Let *E_i_,_j_* represent the log_2_(RSEM + 1) value of the key gene *i* in tumour sample *j*, and *C_i_* represents the corresponding coefficient of the mast cell‐related genes. The risk score MastCell_pca_ was calculated as follows:

$${\text{MastCell}}_{\text{pca}}=\left[\begin{array}{ccc} E_{11} & \cdots & E_{1j}\\ \vdots & \ddots & \vdots \\ E_{i1} & \cdots & E_{\mathrm{ij}} \end{array}\right]{\left[C_{1}\ldots C_{i}\right]}^{T}$$

### 2.6 ssGSEA implementation and clinical response prediction

The enrichment scores of the hallmark genes were evaluated using single‐sample GSEA (ssGSEA) with r package ‘GSVA’ (Hänzelmann *et al.*, 2013). The hallmark gene sets were obtained from MSigDB. Spearman's coefficient analysis was performed to analyse the correlation between prognostic gene signature‐based risk score and each hallmark. The Tumor Immune Dysfunction and Exclusion algorithm was used to predict the clinical response to immune checkpoint blockade (Jiang *et al.*, 2018).

### 2.7 Neural network construction

PyTorch was employed to construct the neural network to predict the immunotherapy response by the mast cell‐related gene signature in python (Version: 3.5) (Paszke *et al.*, 2017). Stochastic gradient descent method and learning rate 0.001 were chosen for the optimizer of the model. Five layers were built with different input and output numbers. Batch normalization was performed in each layer. Dropout function (dropout rate: 0.2) was used in the training process but not in the testing process. Relu function was applied as the activate function. A logistic sigmoid function was used in the output layer. The Python script is provided in Appendix S2.

### 2.8 Random forest algorithm for feature importance ranking

A random forest algorithm was applied to find the most critical mutations associated with the mast cell signature‐based risk score. Briefly, the gene mutation data set (Appendix S3) and mast cell signature‐based risk score were applied to find the most important gene mutations associated with the mast cell signature‐based risk score. First, the ‘ranger’ package was used to find the best hyperparameter in the regression process (Wright and Ziegler, 2015). Then, the ‘randomforest’ package was applied for the construction of the regression model (Liaw and Wiener, 2002). The r code for the analysis in the manuscript is provided in Appendix S4.

## 3 Results

### 3.1 High mast cell abundance in early‐stage LUAD benefits the survival of patients

**Fig. 1:**
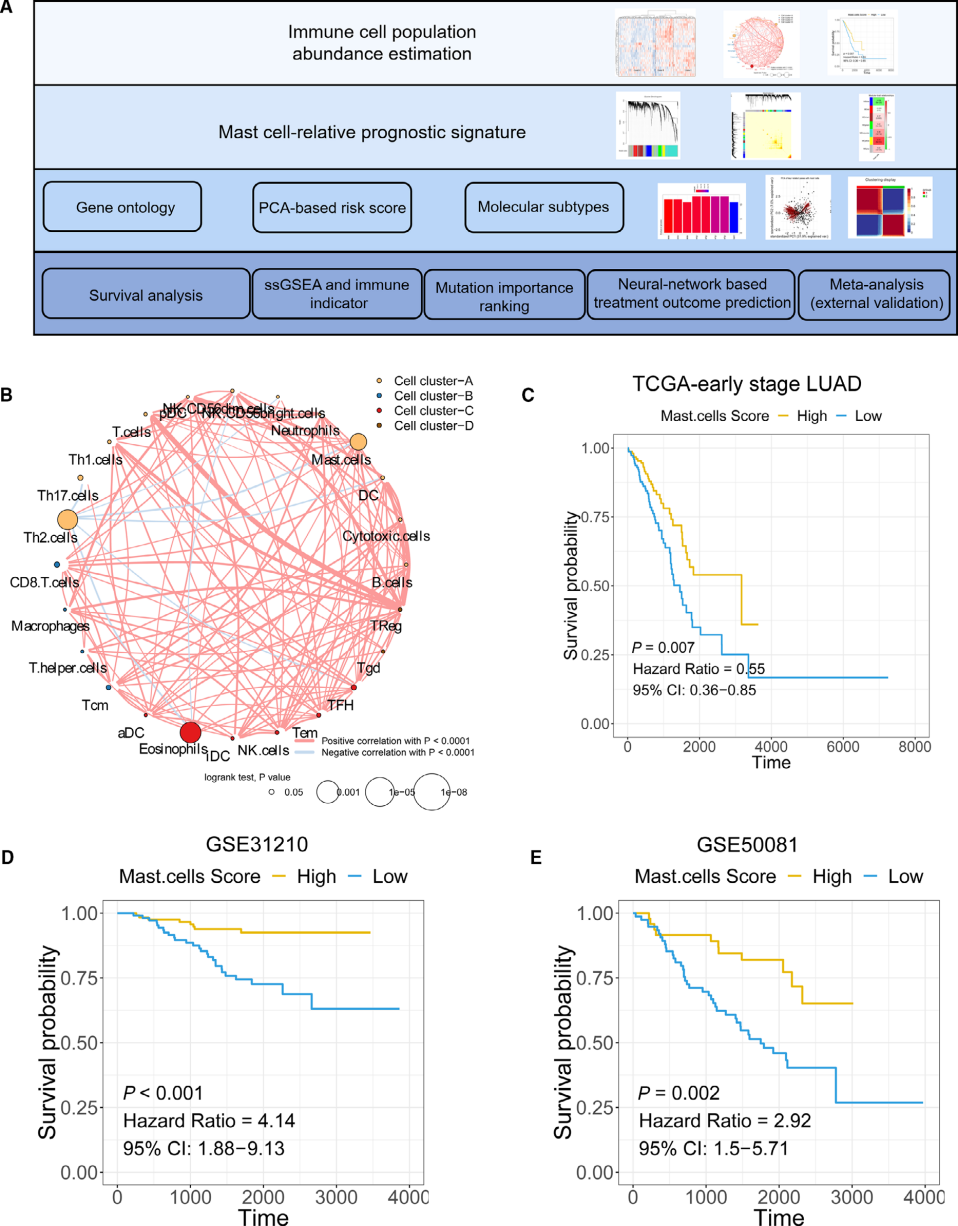
The association between mast cell abundance and clinical outcomes in early‐stage LUAD patients. (A) Schematic diagram of the study design. (B) The correlation among immune cell populations. (C–E) Kaplan–Meier curves for the OS of early‐stage LUAD patients showed that the patients with high mast cell abundance had a favourable outcome compared with the patients with low mast cell abundance in the TCGA, http://www.ncbi.nlm.nih.gov/geo/query/acc.cgi?acc=GSE31210 and http://www.ncbi.nlm.nih.gov/geo/query/acc.cgi?acc=GSE50081 cohorts.

The workflow of the manuscript is shown in Fig. 1A. To illustrate the correlation between mast cells and survival in early‐stage LUAD patients, we first analysed the abundance of immune cell populations in early‐stage LUAD tumour samples. We identified twenty‐two immune cell populations, and the correlations between these populations are shown in Fig. 1B. We found that high mast cell abundance benefited the survival of early‐stage LUAD patients in the TCGA cohorts (Fig. 1C). To further confirm the association between mast cells and the survival of early‐stage LUAD patients, we estimated the abundance of mast cells in two external cohorts (http://www.ncbi.nlm.nih.gov/geo/query/acc.cgi?acc=GSE31210 and http://www.ncbi.nlm.nih.gov/geo/query/acc.cgi?acc=GSE50081). The results showed that high mast cell abundance is associated with prolonged survival of early‐stage LUAD patients, as we observed in the TCGA early‐stage LUAD cohort (Fig. 1D,E).

### 3.2 Identification of a gene signature associated with mast cells

**Fig. 2:**
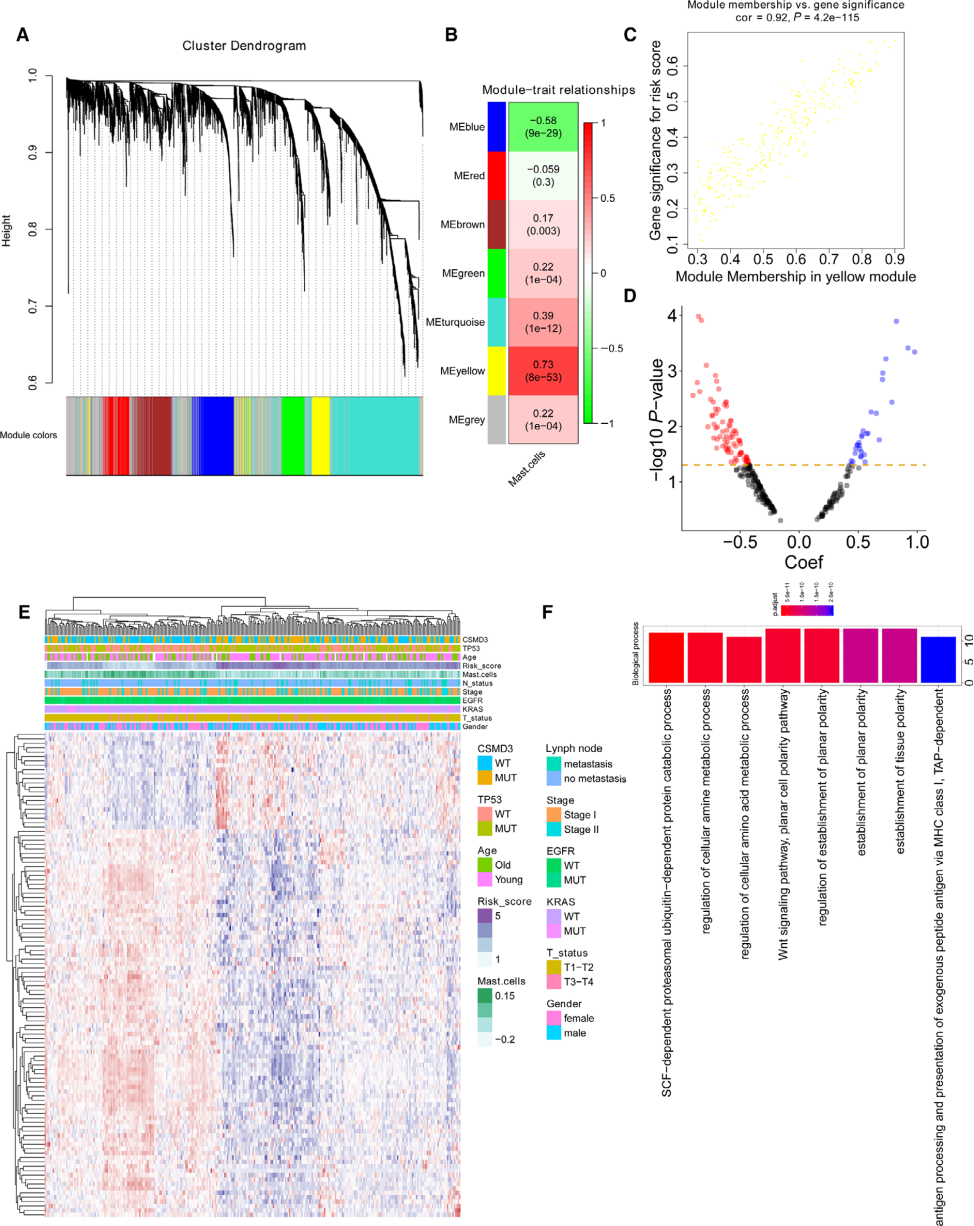
Mast cell‐related gene signature identification. (A) WGCNA was performed to identify seven modules by unsupervised clustering. (B) A total of six modules (nongrey) were identified. The yellow module had the highest correlation (*r* = 0.73, *P* = 8e^−53^) and was considered the most correlated with mast cells. (C) The gene significance and module membership of the genes in the yellow module exhibited a high correlation. (D) A total of 110 mast cell‐related genes were identified among the hub genes extracted from the yellow module. (E) The expression profile of the 110 mast cell‐related genes. (F) GO analysis was performed based on the 110 mast cell‐related genes.

The immune‐related genes were determined with WGCNA. Genes were clustered into seven modules (Fig. 2A). The correlation between the modules and mast cell abundance was calculated by Pearson's correlation coefficient (Fig. 2B). The yellow module showed the highest correlation coefficient with mast cells (cor: 0.73). The plots of module membership and gene significance illustrated a significant correlation for each gene in the yellow module (cor: 0.92; Fig. 2C). Then, each gene in the yellow module was analysed with a univariate Cox regression analysis. We identified 110 genes that were significantly associated with the survival of early‐stage LUAD patients (Fig. 2D). The heat map shows the expression level of the 110 genes (Fig. 2E). The 110 genes were defined as a mast cell‐related gene signature (Appendix S5) in early‐stage LUAD patients. GO analysis revealed that cellular metabolic pathways, WNT signalling, antigen processing and presentation, and other enriched pathways were associated with the mast cell‐related key genes (Fig. 2F).

### 3.3 Molecular subtype identification based on the mast cell‐related gene signature in early‐stage LUAD

**Fig. 3:**
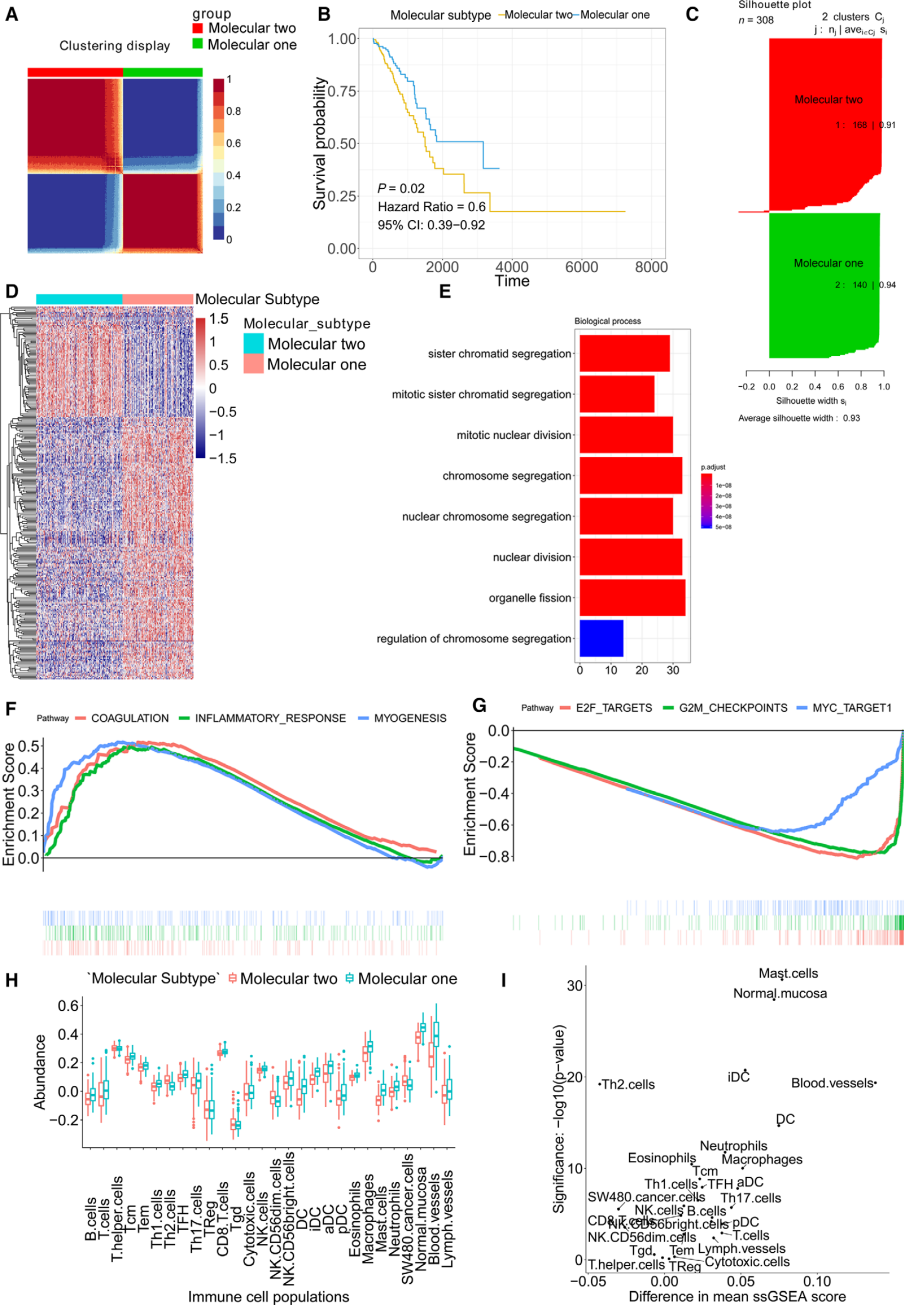
Molecular subtype identification according to the mast cell‐related gene signature. (A) Clustering heat map for intuitively visualizing the effect of sample clustering. (B) Univariate Cox analysis and Kaplan–Meier curves were used to evaluate the survival difference between the two molecular subtypes. (C) Average silhouette width between the two molecular subtypes. (D) The DEGs between two molecular subtypes. (E) GO analysis. (F) Upregulated hallmarks in the GSEA. (G) Downregulated hallmarks in the GSEA. (H) The immune cell population distribution in the subtype 1 and subtype 2. (I) The difference in immune cell population scores and the significances between the subtype 1 and subtype 2.

As we observed, two expression patterns were identified in the expression profiles of mast cell‐related genes from expression heat map of mast cell‐related gene signature. We asked whether the mast cell‐related gene signature could distinguish the molecular subtypes of early‐stage LUAD. Using a combination of gene mutation data sets (genome characteristics) and the expression profiles of mast cell‐related key gene signature (genetic characteristics), we performed molecular subtype identification on early‐stage LUAD patients. Three methods were applied to show the classification effect of the molecular subtypes: (a) a clustering heat map was generated to intuitively visualize the effect of sample clustering (Fig. 3A); (b) univariate Cox and Kaplan–Meier analyses were used to evaluate the significance of the difference in survival profiles between subtypes (HR = 0.59; Fig. 3B); and (b) the average silhouette width, a measure of cluster coherence, was calculated to appraise whether samples were more similar within or across subtypes (Fig. 3C). The results above indicated that mast cell‐related key genes could stratify early‐stage LUAD into two molecular subtypes (subtype 1 and subtype 2) with distinct clinical and molecular characteristics. Tumours of molecular subtype 2 had greater average mast cell densities compared with tumours of molecular subtype 1.

Differently expressed gene analysis was performed to identify the DEGs between the subtype 1 and subtype 2 molecular subtypes. The heat map shows the expression profile of the DEGs (adjusted *P*‐value < 0.05 and log_2_ (FC) > 1.5; Fig. 3D). Then, the DEGs were subjected to GO analysis (Fig. 3E). The results revealed enrichments in cell cycle‐related terms. GSEA was performed on the subtype 1 and subtype 2 of early‐stage LUAD. Upregulated pathways included pathways related to coagulation, inflammatory response and myogenesis in the subtype 1 (Fig. 3F). Downregulated pathways included pathways related to E2F targets, G2M checkpoints and MYC targets in the subtype 1 (Fig. 3G). The immune cell population distribution in the subtype 1 and subtype 2 further illustrated the different tumour immune microenvironments in the two molecular subtypes of early‐stage LUAD (Fig. 3H). Among all immune cell populations, mast cells showed the most significant difference between the subtype 1 and subtype 2 (Fig. 3I).

### 3.4 Neural network‐based model to identifying immunotherapy treatment outcomes

**Fig. 4:**
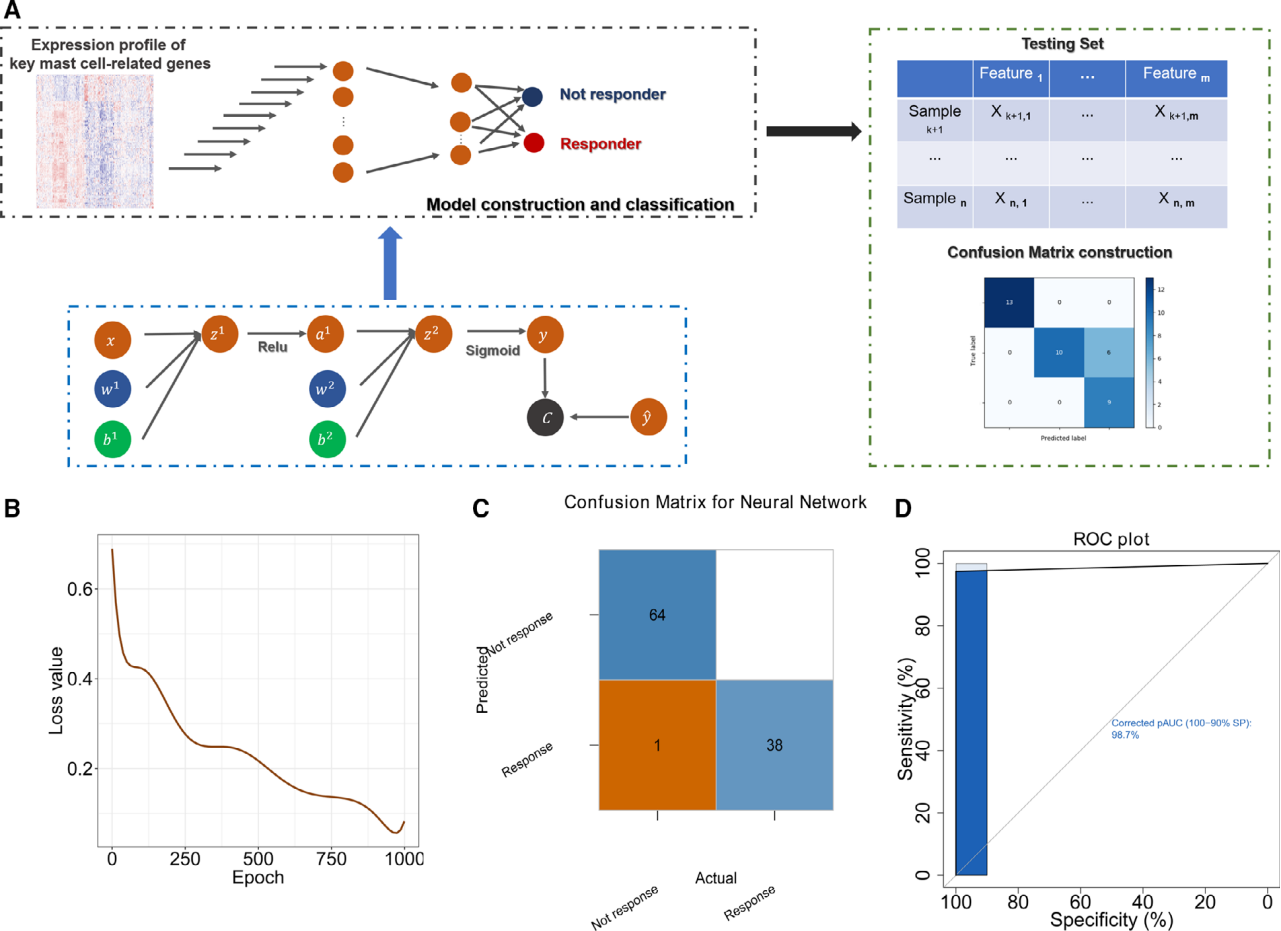
Neural network‐based framework construction with the mast cell‐related gene signature. (A) Schematic diagram of the neural network. (B) The loss value in each epoch during training process in the validation cohort. (C) The confusion matrix in the testing cohort validated the accuracy of the network's prediction capacity. (D) The ROC plot in the testing data set validated the accuracy of the network's prediction capacity.

To further utilize the mast cell‐related gene signature we identified, we built a neural network‐based framework to predict which patient would respond to immunotherapy according to mast cell‐related key genes. The detailed code is provided in Appendix S2. Figure 4A illustrates a diagram of the neural network. Briefly, the early‐stage LUAD data set was divided into training and testing data sets. We constructed the neural network with the mast cell‐related gene signature by the training data set. The test data set was applied to evaluate the accuracy of the neural network. With the increased epoch number for training, the loss value of the model in the testing set decreased (Fig. 4B). The confusion matrix showed only one sample was recognized wrongly in the testing set (Fig. 4C). The receiver operating characteristic (ROC) curve illustrated a high accuracy rate with the area under the curve reaching 98.7% (Fig. 4D).

### 3.5 Mast cell‐related signature predicts the prognosis and clinical outcome of early‐stage LUAD patients

**Fig. 5:**
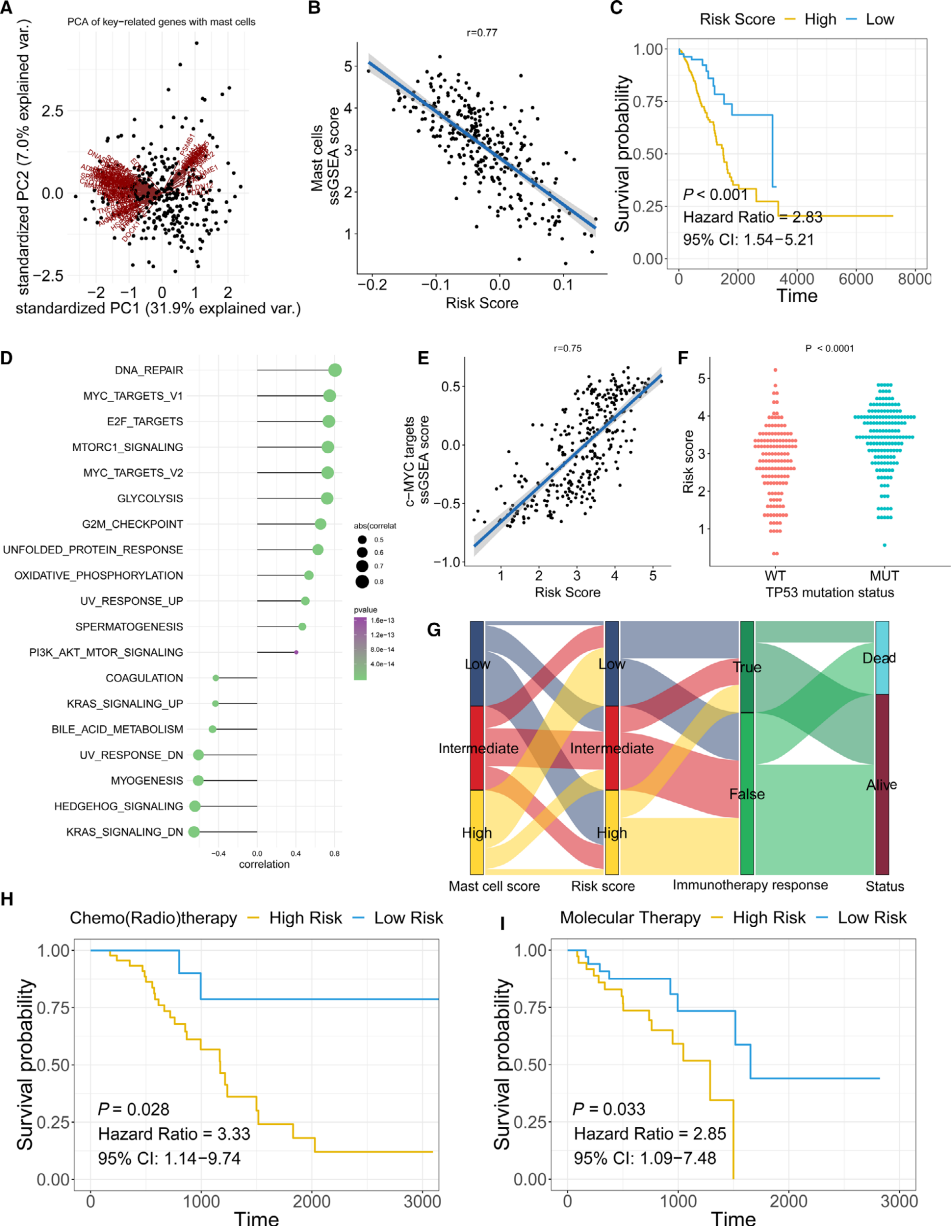
Mast cell‐related signature‐based risk score calculation and the potential mechanism underlying the mast cells in early‐stage LUAD. (A) PCA of the key mast cell‐related genes. (B) The correlation between the prognostic signature‐based risk score and the mast cell ssGSEA score in early‐stage LUAD patients. (C) Univariate Cox analysis and Kaplan–Meier curves showed prolonged survival in patients with low‐risk scores compared with patients with high‐risk scores. (D) The correlation between the ssGSEA score of each hallmark gene and the risk score. (E) The correlation between the risk score and ssGSEA score in early‐stage LUAD patients. (F) The risk score distribution in patients with wild‐type or mutated TP53. *P*‐value was calculated with Mann–Whitney *U*‐test. (G) A Sankey plot was used to reveal the correlation between mast cell scores, prognostic signature‐based risk scores, immunotherapy response and clinical outcome. (H, I) Patients who received adjuvant therapies, including chemo(radio)therapy and targeted therapy, with low‐risk scores, exhibited prolonged overall survival.

The mast cell‐related gene signature was employed to calculate a prognostic risk score. The risk score MastCell_pca_ was calculated for each patient using the PCA method. Figure 5A shows the first principal component (PCA1) score for each key mast cell‐related gene. MastCell_pca_ was calculated with the expression level of each gene and the PCA1 score. The results showed a highly negative correlation between MastCell_pca_ and mast cell abundance, which further confirmed the correlation between the mast cell‐related key genes and mast cells (Fig. 5B). The Kaplan–Meier plot revealed that patients with a low‐risk score had a better prognosis than patients with a high‐risk score (Fig. 5C). ssGSEA results revealed a high association of DNA repair and the c‐MYC pathway with the mast cell‐related risk score (Fig. 5D). TP53 mutations can regulate the activation of c‐MYC pathway (Frazier *et al.*, 1998). Due to the high correlation between the c‐MYC pathway and the mast cell‐related risk score, we selected TP53 mutation as an example given its role in regulating the activation of c‐MYC pathway (Frazier *et al.*, 1998) and analysed the mast cell‐related risk score in TP53‐mutated and wild‐type patients (Fig. 5E,F). The results showed a high‐risk score in the TP53‐mutated patients.

Furthermore, patients with high mast cell abundance had a low mast cell‐related risk score and responded to immunotherapy (Fig. 5G). In patients who received chemo(radio)therapy and molecular therapy, the patients with low mast cell‐related risk scores had better survival outcomes than those with high mast cell‐related risk scores (Fig. 5H,I).

### 3.6 The association between mast cell‐related signature and gene mutation in early‐stage LUAD

**Fig. 6:**
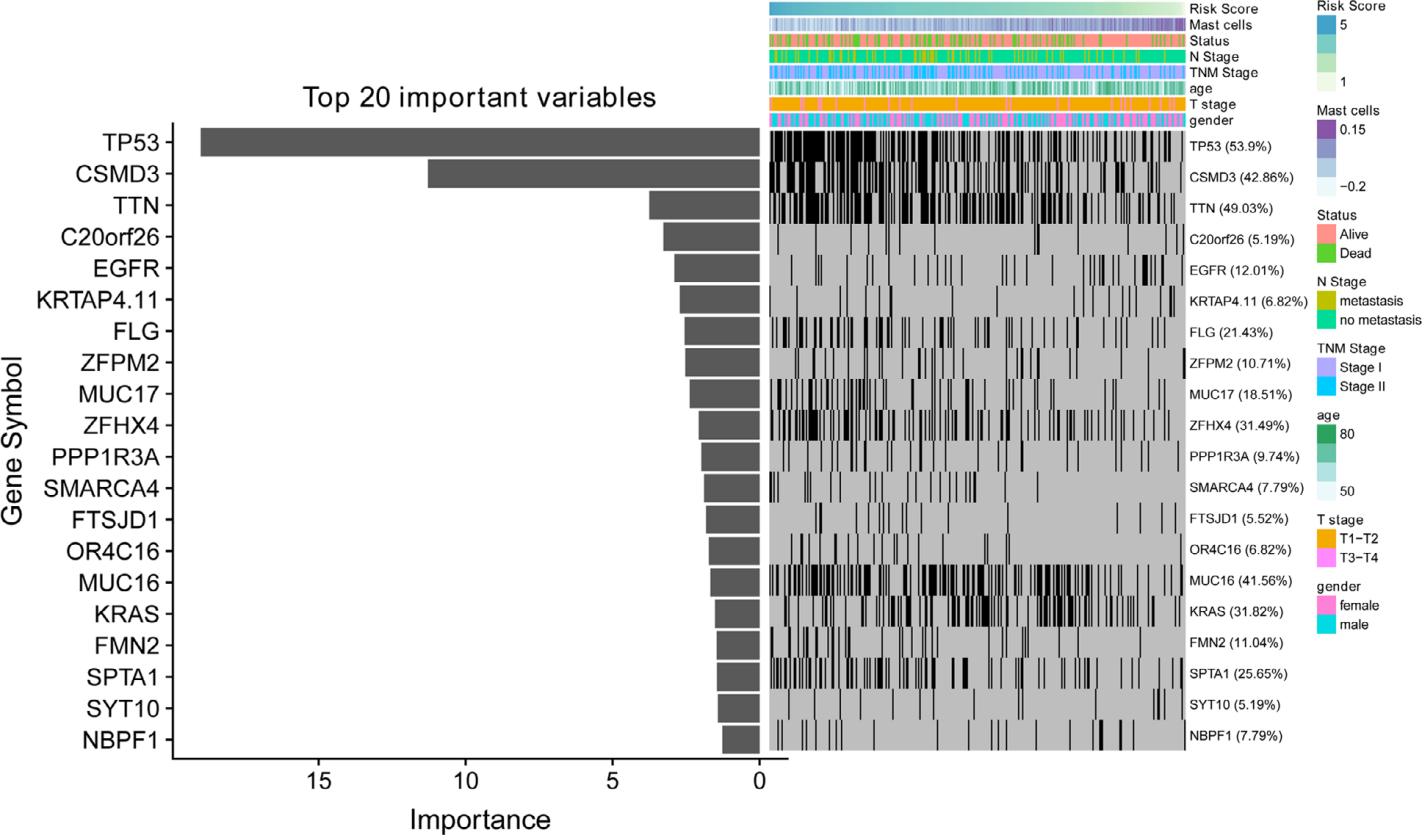
Association of the immune signature with early‐stage LUAD gene mutations. The distribution of gene mutations correlated with the prognostic signature‐based risk score. TP53 was the most important mutation according to the importance of ranking.

The random forest algorithm was employed to determine the importance of gene mutations associated mast cell‐related risk score (Fig. 6). The results revealed that TP53 and CSMD3 were the most important gene mutations associated with the mast cell‐related risk score. The patients with TP53 mutations had significantly higher mast cell‐related risk scores than the patients without TP53 mutations.

### 3.7 External validation and meta‐analysis

**Fig. 7:**
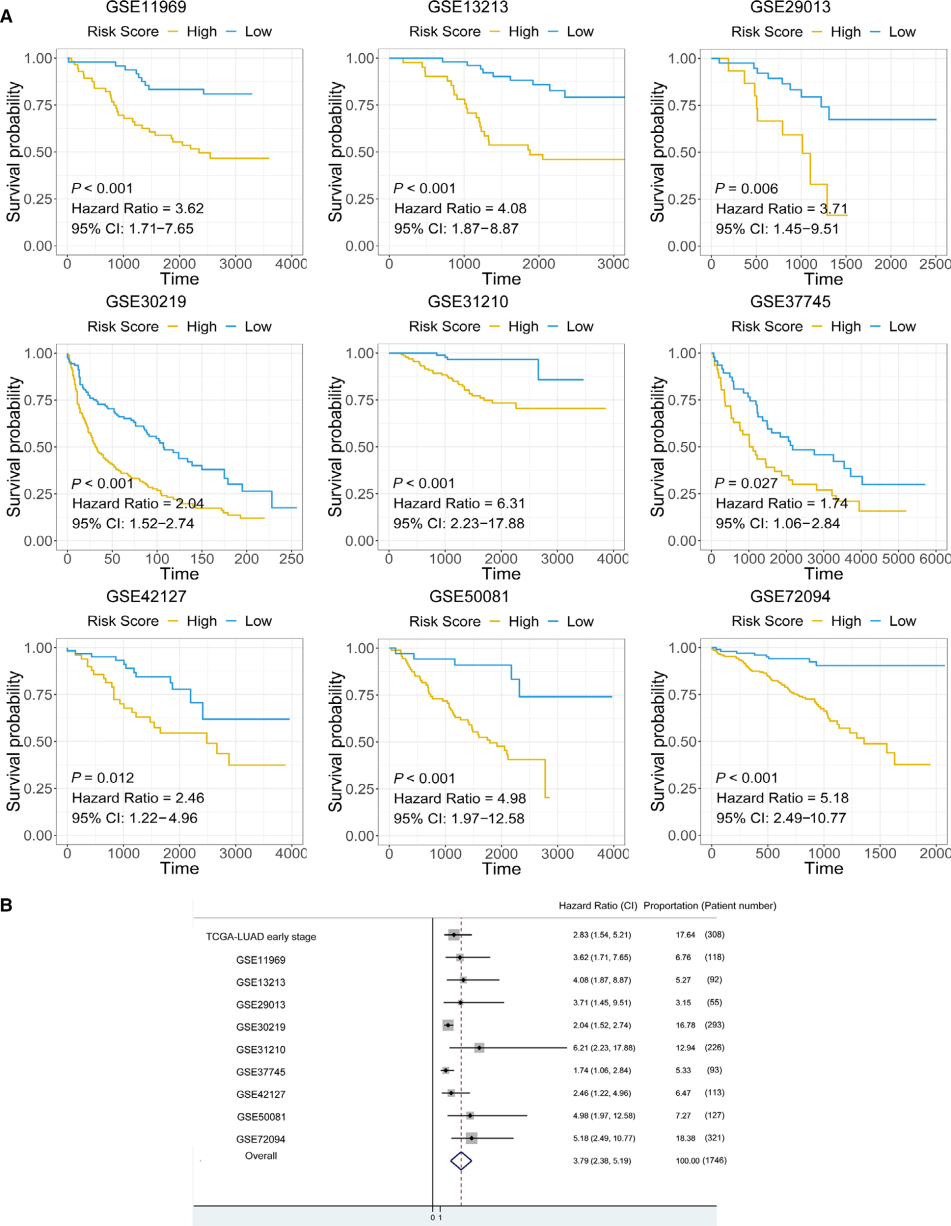
Meta‐analysis and external validation of the prognostic value of the mast cell‐related signature. (A) Detailed information for the nine external validation cohorts. (B) A meta‐analysis revealed the overall prognostic value of the mast cell‐related signature.

Nine external cohorts were used to confirm the association between the mast cell‐related gene signature and survival outcomes in early‐stage LUAD patients. The detailed information for each cohort is shown in the Kaplan–Meier plot (Fig. 7A). A meta‐analysis was performed with a random‐effects model, and the results showed that patients with a high mast cell‐related risk score had poor survival outcomes in the overall data set (HR = 3.79; Fig. 7B).

## 4 Discussion

Previous studies have investigated the relationship between immune cell populations and the clinical outcomes of cancer patients (Bao *et al.*, 2019b; Bindea *et al.*, 2013; Chung *et al.*, 2017; Homma *et al.*, 2014). The heterogeneity of immune cell populations in different cancer types leads to a complicated immune network in the tumour microenvironment and differentially influences tumour initiation and progression. As a major component of the immune microenvironment in tumour tissues, mast cells may play a pro‐tumorigenic or antitumorigenic role by releasing different mediators (Varricchi *et al.*, 2017). For instance, angiogenic and lymphangiogenic factors secreted by mast cells promote tumour angiogenesis and lymphangiogenesis (Detoraki *et al.*, 2010; Detoraki *et al.*, 2009; Theoharides *et al.*, 2010). Several matrix metalloproteinases released by mast cells regulate the digestion of tumour extracellular matrix and favour the distant metastasis of cancer cells (Baram *et al.*, 2001). Specifically, the activation of MYC triggers rapid recruitment of mast cells to the tumour site to promote tumour expansion in pancreatic cancer. MYC directly commandeers and instructs tissue remodelling, angiogenesis and inflammation by activation of mast cells (Soucek *et al.*, 2007). Mast cells release tryptase AB1 and interleukin‐1β, which in turn induced pleural vasculature leakiness and triggered NF‐κB activation in pleural tumour cells, thereby fostering pleural fluid accumulation and tumour growth (Giannou *et al.*, 2015). In contrast, mast cells can exhibit antitumour activity directly through tumour cell cytotoxicity mediated by TNF‐α and ROS or indirectly through the release of interleukin‐9 and heparin and the stimulation of dendritic cell maturation (Varricchi *et al.*, 2017). The complicated roles of mast cells allow them to play different functions in different cancer types and stages.

Regarding the impact of mast cells on LUAD patient outcomes, several contradictory studies have been published. One study revealed that mast cells correlated with angiogenesis and poor outcome in stage I LUAD (Baram *et al.*, 2001). Another study has revealed KIT‐competent mast cells fuel KRAS‐mutant LUAD formation, growth and metastasis by providing interleukin‐1β and are associated with LUAD progression (Lilis *et al.*, 2019). However, one research indicated that only mast cells were found by univariate analysis to be associated with better prognosis in LUAD (Kurebayashi *et al.*, 2016). Although the results of these studies are quite different with respect to the prognostic value of mast cells, previous studies have shown that mast cell infiltration is more intensive in low‐grade histologic subtypes than in high‐grade subtypes (Carlini *et al.*, 2010). Understanding the potential mechanism and roles of mast cells in early‐stage LUAD may be helpful for the development of immunotherapy. Thus, in this study, we analysed the potential role and mast cell‐related genes in early‐stage LUAD. The abundance of mast cells was estimated in several cohorts. Cox regression was performed to identify the prognostic value of mast cells in early‐stage LUAD. WGCNA was employed to identify the mast cell‐related gene signature. Molecular subtypes (subtype 1 and subtype 2) were identified according to the mast cell‐related gene signature in a mutation data set of early‐stage LUAD. A neural network‐based framework was constructed to predict the immunotherapy outcome of early‐stage LUAD patients according to the mast cell‐related gene signature. A mast cell‐related risk score MastCell_pca_ was calculated by the expression levels of mast cell‐related gene signature using the PCA method. ssGSEA was performed to identify the potential molecular mechanism associated with the mast cell‐related prognostic signature. The association between gene mutations and the risk scores was identified by a random forest algorithm. A meta‐analysis was performed to validate the mast cell‐related signature in external cohorts.

In our analysis, we revealed that a high abundance of mast cells was associated with prolonged survival in early‐stage LUAD patients. Two external cohorts confirmed this conclusion. The differences and controversial conclusions in different studies (Baram *et al.*, 2001; Kurebayashi *et al.*, 2016; Lilis *et al.*, 2019) may be due to the mixture of activated and resting mast cells. The function of activated mast cells may be masked by the resting mast cells. Therefore, it is essential to analyse the activated and resting mast cells separately. In an alternative way, we employed the following workflow to identify the potential mechanisms and genes associated with mast cells.

First, the immune‐related genes were clustered into several modules by unsupervised clustering. The yellow module was identified as the most important module correlated with mast cells according to Pearson's correlation coefficient. The mast cell‐related gene signature was obtained from the yellow module. The genes in the mast cell‐related gene signature were highly associated with the mast cell density in early‐stage LUAD. According to the mast cell‐related gene signature and genome characteristics, the early‐stage LUAD tissues were stratified into two molecular subtypes (subtype 1 and subtype 2). Interestingly, the GO analysis and GSEA both indicated enrichments in cell cycle and c‐MYC‐related pathways in the subtype 2. Thus, we concluded the potential involvement of mast cells in the c‐MYC pathway in early‐stage LUAD. One previous study has demonstrated the important roles of mast cells in MYC activation and the potential tumour expansion promoted by mast cells in pancreatic cancer. MYC is a highly pleiotropic transcription factor whose aberrant activation links tightly with tumour progression, including both cell‐intrinsic proliferation and extracellular microenvironment alterations such as tissue remodelling, angiogenesis and invasion (Gabay *et al.*, 2014). Aberrant MYC activities induce the dysregulated expression of a chemokine‐encoding gene cluster, therefore chemoattracting mast cells into the islets of pancreatic cancer (Soucek *et al.*, 2007). In consistent with the pancreatic cancer study, the transcriptomic and downstream analyses underscore the importance of mast cell in MYC activation in early‐stage LUAD. Moreover, the differences in mast cells between the two subtypes were the most significant of all the immune cell populations studied, which confirmed the relationship between the mast cell‐related gene signature and mast cell abundance. The mast cell‐related gene signature may represent targets for further study to aid in the understanding of the mechanism of mast cells in early‐stage LUAD.

To further utilize the mast cell‐related gene signature, we built a neural network‐based framework to predict response to immunotherapy. The confusion matrix and ROC plot confirmed the accuracy of the network's prediction capability. Hence, we were able to apply the expression profile of the mast cell‐related genes to predict the response to immunotherapy using the neural network framework.

In the next step, we calculated the risk score MastCell_pca_ according to the expression level of the gene signature for each patient. ssGSEA revealed a significant correlation between DNA repair, the c‐MYC pathway and the signature‐based risk score. The ssGSEA results further confirmed the results from the canonical GSEA of the molecular subtypes. c‐MYC stimulates the expression of target genes that play important roles in cell proliferation, growth arrest and apoptosis in lung cancer cells (Dang *et al.*, 2006; Tong *et al.*, 2004). Additionally, we further identified TP53 as the most critical mutation associated with the mast cell‐related signature. Dysregulation of the c‐MYC pathway induces the expression of endogenous TP53. As a cellular gatekeeper, TP53 plays crucial role in cell cycle arrest and apoptosis (Mogi and Kuwano, 2011). The close link between the mast cell‐related signature, the c‐MYC pathway and TP53 mutation in our analysis may highlight the roles of mast cells in early‐stage LUAD. However, as we suggested, analysing the activated and resting mast cells separately would be a promising way for understanding the molecular mechanism of mast cells in early‐stage LUAD. The mast cell‐related gene signature we obtained may therefore prove useful information for further study in this field.

The mast cell‐related signature also served as a promising marker to predict the survival of early‐stage LUAD patients. We performed a meta‐analysis by combining nine cohorts. The results revealed in both each cohort and the meta‐analysis that the mast cell‐related signature stratified the survival of patients with high and low signature‐based risk scores. The results above also confirmed the pivotal roles of mast cells in early‐stage LUAD.

A problem with the mast cell‐related signature of early‐stage LUAD as shown is that only in silico analysis is performed. Experimental studies are required to further elucidate the biological functions underlying the mast cell‐related signature in early‐stage LUAD. Large, well‐designed prospective population‐based studies should be conducted to investigate the complex role of mass cell and testify our results on mast cell‐related signature.

## 5 Conclusion

In this study, we depicted the correlation between mast cell populations and prognosis in early‐stage LUAD patients. A mast cell‐related gene signature was identified. A novel molecular subtype classification and a mast cell‐related gene signature‐based neural network were built to help understanding of mast cell activities in early‐stage LUAD and aid in the development of immunotherapy for early‐stage LUAD patients. Potential pathways associated with the mast cell‐related gene signature provide new directions for determining novel mechanisms in mast cells in early‐stage LUAD. The results above may facilitate personalized medicine for early‐stage LUAD patients.

## Conflict of interest

The authors declare that the research was conducted in the absence of any commercial or financial relationships that could role as a potential conflict of interest.

## Author contributions

XB, YW and RS conceived and designed the experiments. XB and RS performed the analysis. XB and YW wrote the paper. XB, TZ, RS and YW reviewed the draft. All authors read and approved the final manuscript.

## Supporting information


**Appendix S1.** Patient clinical information.

**Appendix S2.** The Python script for building the neural network‐based framework.

**Appendix S3.** The gene mutation data set.

**Appendix S4.** The r script.

**Appendix S5.** The gene list of the mast cell‐related gene signature.

## Data Availability

The data sets supporting the conclusions of this article are available in the Xena Browser (https://xenabrowser.net/) repository.
