# Supplementary material for: Mast cell‐based molecular subtypes and signature associated with clinical outcome in early‐stage lung adenocarcinoma
Source: Mol Oncol. 2020 Apr 1;14(5):917–32. doi: 10.1002/1878-0261.12670 (PMC7191192; doi:10.1002/1878-0261.12670)
Supplement: Supplementary file 5 — Appendix S5. The gene list of the mast cell‐related gene signature. [file MOL2-14-917-s005.docx]

| **Gene symbol** |
| --- |
| \| *ITPKB* \| \| --- \| \| *LRIG1* \| \| *PSMC6* \| \| *PSMB1* \| \| *FANCL* \| \| *FADD* \| \| *PGM5* \| \| *PSMA5* \| \| *BTG2* \| \| *PSMC4* \| \| *PCDH11X* \| \| *EDN3* \| \| *TNFRSF19* \| \| *CASS4* \| \| *LRRN3* \| \| *ART4* \| \| *NFATC3* \| \| *NRXN1* \| \| *PSMA3* \| \| *ITGA9* \| \| *DNASE1L3* \| \| *FNDC3A* \| \| *CERK* \| \| *IHH* \| \| *PSMA1* \| \| *ANGPT1* \| \| *CLDN12* \| \| *TSLP* \| \| *FBLN5* \| \| *PRKCE* \| \| *SATB1* \| \| *DOCK1* \| \| *C7* \| \| *WNT11* \| \| *C1QTNF7* \| \| *MAMDC2* \| \| *IL33* \| \| *SCARA5* \| \| *SPARCL1* \| \| *CFL1* \| \| *IFT172* \| \| *CNTN3* \| \| *IGSF10* \| \| *BCL6B* \| \| *LATS2* \| \| *FZD4* \| \| *MAOB* \| \| *SOX18* \| \| *LTBP4* \| \| *ROBO2* \| \| *PSMC3* \| \| *HGF* \| \| *AGTR1* \| \| *FIGF* \| \| *HCG11* \| \| *SMAD9* \| \| *ANGPTL1* \| \| *CDHR3* \| \| *SFTPD* \| \| *GATA2* \| \| *MFAP4* \| \| *PDCD5* \| \| *NEGR1* \| \| *ARID5B* \| \| *NEDD9* \| \| *EDN1* \| \| *PSMA2* \| \| *DAD1* \| \| *CNTFR* \| \| *GHR* \| \| *RORA* \| \| *PCDH15* \| \| *RABEPK* \| \| *RNF7* \| \| *TCF21* \| \| *ESAM* \| \| *A2M* \| \| *HSPB11* \| \| *FTO* \| \| *AKAP13* \| \| *ADAMTS8* \| \| *TCF7L1* \| \| *GAB2* \| \| *PSMC1* \| \| *NCAM1* \| \| *FREM2* \| \| *NGFR* \| \| *PSMC2* \| \| *MTSS1* \| \| *HSPG2* \| \| *SH3GL2* \| \| *ARC* \| \| *DUOX1* \| \| *LPL* \| \| *PBX1* \| \| *CASP12* \| \| *SLC14A1* \| \| *DARC* \| \| *VDAC1* \| \| *TUB* \| \| *PSMB7* \| \| *CLDN18* \| \| *CHRM1* \| \| *NME1* \| \| *PTPRQ* \| \| *NDN* \| \| *MASP1* \| \| *KALRN* \| \| *SLIT3* \| \| *CD34* \| |
